# Differential effects of *SORL1* deficiency on the endo-lysosomal network in human neurons and microglia

**DOI:** 10.1098/rstb.2022.0389

**Published:** 2024-04-08

**Authors:** Swati Mishra, Suman Jayadev, Jessica E. Young

**Affiliations:** ^1^ Department of Laboratory Medicine and Pathology, University of Washington, Seattle, WA 98109, USA; ^2^ Deparment of Neurology, University of Washington, Seattle, WA 98109, USA; ^3^ Institute for Stem Cell and Regenerative Medicine, University of Washington, Seattle, WA 98109, USA

**Keywords:** SORL1, Alzheimer's disease, endosomal trafficking, neurons, microglia

## Abstract

The endosomal gene *SORL1* is a strong Alzheimer's disease (AD) risk gene that harbours loss-of-function variants causative for developing AD. The *SORL1* protein SORL1/SORLA is an endosomal receptor that interacts with the multi-protein sorting complex retromer to traffic various cargo through the endo-lysosomal network (ELN). Impairments in endo-lysosomal trafficking are an early cellular symptom in AD and a novel therapeutic target. However, the cell types of the central nervous system are diverse and use the ELN differently. If this pathway is to be effectively therapeutically targeted, understanding how key molecules in the ELN function in various cell types and how manipulating them affects cell-type specific responses relative to AD is essential. Here, we discuss an example where deficiency of SORL1 expression in a human model leads to stress on early endosomes and recycling endosomes in neurons, but preferentially leads to stress on lysosomes in microglia. The differences observed in these organelles could relate to the unique roles of these cells in the brain as neurons are professional secretory cells and microglia are professional phagocytic cells. Experiments to untangle these differences are fundamental to advancing the understanding of cell biology in AD and elucidating important pathways for therapeutic development. Human-induced pluripotent stem cell models are a valuable platform for such experiments.

This article is part of a discussion meeting issue ‘Understanding the endo-lysosomal network in neurodegeneration’.

## 1. Main

Endo-lysosomal dysfunction is apparent in Alzheimer's disease (AD) and emerging genetic studies repeatedly implicate genes encoding endosomal proteins as associated with increased AD risk. One of the earliest pathologies seen in AD brain tissue are enlarged endosomes and indicators of faulty lysosomes, which is suggestive of high stress on the system [1–7]. In AD, both early onset autosomal dominant AD genes and an increasing number of genes associated with late-onset AD risk are associated with the endo-lysosomal network (ELN). Presenilins, especially presenilin 2 and pathogenic variants of presenilin 1, are localized to endo-lysosomal vesicles and variants in *PSEN1/2* disrupt lysosome function, alter autophagy and result in enlarged early endosomes [8,9]. In particular, presenilin 2 localization is highly restricted to late endosomes and lysosomes, leading to a distinct intracellular pool of pathogenic A*β* [10]. Mouse models either deficient in or with AD-associated mutations in presenilin 1 have shown striking abnormalities in lysosomal and autolysosome acidification and proteolysis, due to mis-targeting of v-ATPase subunits to lysosomes [11]. This impairment in lysosome acidification can disrupt lysosomal calcium homeostasis [12]. Abnormal endosomes in *PSEN1* mutant mice also lead to deficits in motor protein function, which ultimately results in altered transport of endosomes to the soma and swollen axons [13]. Autosomal dominant variants in the Amyloid Precursor Protein gene, *APP*, lead to enlarged early endosomes in human-induced pluripotent stem cell (hiPSC)-derived neurons and cortical organoids, can alter interactions between APP and BACE1 in endocytic compartments, affect intracellular sorting and ultimately increase amyloidogenic processing [14,15]. Endo-lysosomal dysfunction can affect multiple central nervous system (CNS) cell types in these models. Mice harbouring the *APP* Swedish (K670N/M671L) mutation accumulate intracellular deposits of A*β* and *APP* *β* C-terminal fragments (*β*CTFs) in neurons early in their disease state, a phenomenon that is replicated in AD patient neurons [16]. Novel *APP* knock-in mouse models with familial AD (FAD) *APP* mutations also show enlarged lysosomes, increased intracellular A*β* accumulation associated with lipid dysregulation and immune perturbations in microglia [17].

Late-onset risk genes such as *SORL1, BIN1, PICALM, CD2AP* and others play various roles in ELN trafficking [18–22]. BIN1 and RAB5 proteins interact in a complex with RIN3 (Ras and Rab Interactor 3) to regulate endocytosis and trafficking in neurons and this complex may regulate APP cleavage in endosomes [23]. Human neuronal *BIN1* isoforms expressed in *Drosophila* photoreceptor neurons induce a blockade of endosomal trafficking and neurotoxicity, while *BIN1* knockout (KO) leads to smaller early endosomes in hiPSC-derived neurons [24]. Recent studies have also cumulatively shown that decreased expression of *SORL1* impacts the endo-lysosomal pathway. Specifically, full loss and haploinsufficiency of *SORL1* lead to enlarged early endosomes in hiPSC-derived neurons and haploinsufficiency of *SORL1* also causes endosome enlargement in neurons of mini-pigs [25–27]. In the mini-pig model, decreased *SORL1* expression is also associated with elevated A*β* and tau levels in the CSF of these animals. Furthermore, complete loss of *SORL1* alters endosomal recycling, autophagy and lysosome function [26,28].

The above studies highlight the complexity of the endo-lysosomal network (ELN), which consists of intracellular, dynamic, membranous organelles that transport various cellular cargo (reviewed in [29]). The ELN is essential for all cell types of the CNS, yet each unique cell type uses cellular trafficking differently [30]. For example, in neurons, the ELN is needed for the internalization and signal transduction of neurotrophic factors and receptors [31], recycling and re-insertion into the plasma membrane of components necessary for the neuronal synapse [32,33] and for efficient degradation of aggregate-prone proteins such as phosphorylated tau [34]. Meanwhile, in microglia, dysfunction of the ELN can have multiple impacts on innnate immune pathways in brain, which can predispose to or exacerbate neurodegeneration [35–39]. The endo-lysosome manages multiple cellular processes critical to microglial cell function, including transport and degradation of cargo, cytokine levels and receptor recycling, and serves as a platform for signalling [40,41]. Therefore, a significant challenge remains: how do we accurately define the role of AD associated genes in the cell-type specific functionality of this network and test how ELN phenotypes may be modified to benefit neural cell function? Understanding how these mechanisms are impaired early in AD is critical for the development of new therapeutics that will improve and potentially reverse early disease progression.

In this *Opinion* *piece*, we will focus on how loss of the endosomal receptor *SORL1* (protein name SORL1 or SORLA) contributes to ELN stress in AD models. SORL1 was originally identified as a member of the low density lipoprotein (LDL) receptor family. SORL1 is now classified as one of five mammalian sorting receptors called VPS10 receptors [42–46]. SORL1 binds APP and protects it from amyloidogenic processing [47] and loss of SORL1 is observed in neurons in sporadic AD (sAD) brains [48,49,50]. Genetic studies have consistently implicated *SORL1* with increased AD risk [51–53]. In 2007, based on biological data linking the multiprotein sorting complex retromer to VPS10 proteins [54,55], a candidate gene study identified two *SORL1* haplotypes that were associated with increased AD risk in several population groups [53]. Exome sequencing studies in 2012 identified rare coding variants in *SORL1* in families with early-onset AD but without known mutations in *APP* or *PSEN1/2* [56]. Subsequent larger exome studies have revealed multiple coding variants in many domains of the protein with varying degrees of pathogenicity [57]. Of these coding variants, those leading to premature termination codons and haploinsufficiency appear to be causative for AD [57,58]. Genome-wide association studies (GWAS) have also associated *SORL1* as a susceptibility locus in sAD and this association has been consistently replicated [51,52]. *SORL1* is also directly implicated in immune response pathways. It binds interleukin 6 (IL-6) to mediate its cellular uptake as well as transmembrane and soluble IL-6 receptor (IL-6R) to regulate IL-6 signalling in astrocytes [59]. SORL1 has also been shown to regulate monocyte motility [60], a key component to the innate immune response to injury.

The complexity of the ELN and the necessity to understand early cellular events in AD pathogenesis support the use of hiPSC models to elucidate key molecular events. hiPSCs have been used for over a decade as a model to combine the strengths of an *in vitro* system with uniquely human components. Nearly every cell type of the CNS and the periphery can be differentiated from hiPSCs, the cells are easily genetically manipulatable using CRISPR/Cas9 technology, characteristics of individual patient genomes are maintained in differentiated cells [61] and these cells are widely used in drug testing and screening [62,63]. We have used hiPSC models to elucidate molecular phenotypes in hiPSC-neurons in cells with common SORL1 variants [64], as well as to show that small molecules that stabilize retromer, a multiprotein sorting complex of which SORL1 is an adaptor protein, can reduce pathogenic tau phosphorylation in an amyloid-independent manner [65]. Based on this work, we have undertaken studies to further understand the cell-type specific role of SORL1 in neurons and microglia and to discern how reduction in SORL1 expression can differentially affect ELN phenotypes in these two cell types.

**Figure 1.**
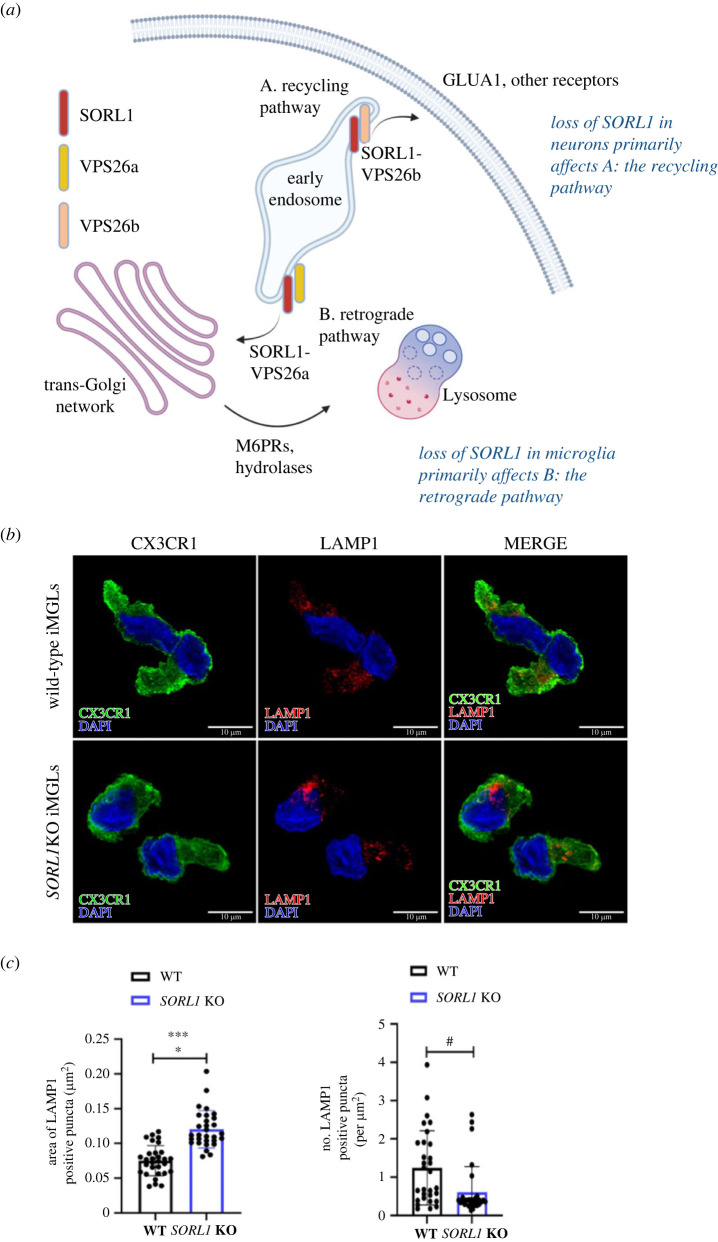
Model of the differential effects of SORL1 deficiency in neurons and microglia. (*a*) SORL1 interacts with two isoforms of VPS26. In neurons, VPS26b is preferentially expressed and with SORL1, traffics cargo to the recycling pathway. In microglia, the ubiquitous isoform VPS26a is highly expressed and with SORL1, traffics cargo to the retrograde pathway. Panel created with BioRender.com. (*b*) Loss of SORL1 causes lysosomal enlargement and decreases LAMP1 + puncta in microglia-like cells (iMGLs). (*c*) Quantification of lysosome size. Data represented as mean ± s.d. and analysed using parametric two-tailed unpaired *t* test. Significance was defined as a value of *p < 0.05, **p < 0.01, ***p < 0.001 and ****p < 0.0001, n.s. = not significant. DAPI, 4',6-diamidino-2-phenylindole.

We have generated an isogenic series of WT, SORL1KO, SORL1 haploinsufficient (SORL1+/-) and SORL1 variant (AD-associated missense variants) hiPSC lines [27,28,66]. In early studies with these cell lines, we observed that full loss of SORL1 leads to enlarged early endosomes in hiPSC-derived cortical neurons [27], a phenotype reminiscent of early cellular pathology first documented in post-mortem AD neurons several decades ago [5]. We further demonstrated that SORL1KO neurons also have enlarged recycling endosomes and significant defects in endosomal recycling, which manifests in reduction of key components of synaptic receptors such as GLUA1, a subunit of the excitatory AMPA receptor complex, on the cell surface [28]. In turn, this impacts the firing of action potentials in SORL1KO neurons [28]. These data correlate nicely with work in mice showing that loss of the retromer component VPS26b also leads to reduction in GLUA1 on the neuronal surface and results in changes in neuronal physiology [67]. The VPS26 protein has two isoforms, with VPS26b preferentially expressed in neurons and VPS26a preferentially expressed in non-neuronal cells, including microglial-like cells [67]. SORL1 and VPS26a/b interact with each other as SORL1 is the adaptor protein between VPS26 and retromer cargo such as APP [68]. Interestingly, in VPS26b-deficient mice, levels of SORL1 are also greatly reduced [67]. Together, these studies suggest that the SORL1-retromer pathway plays distinct roles in neurons versus non-neuronal cells. In neurons, SORL1–VPS26b has a stronger role in regulating endosomal recycling for key neuronal components, such as synaptic proteins. In non-neuronal cells, such as glia, the SORL1–VPS26a pathway plays a stronger role in trafficking cargo through the retrograde pathway from the trans-Golgi network (TGN) to the lysosome (figure 1*a*).

To test this idea, we have analysed endosome and lysosome size in differentiated neurons (hiPSC-Ns) and microglial-like cells (iMGLs), which we have generated from our gene-edited cell lines. Interestingly, we observed enlarged endosomes in hiPSC-Ns, but not in iMGLs [27]. We performed subsequent studies to show that in SORL1-deficient iMGLs there are significantly enlarged lysosomes as well as a reduction in the number of lysosomes (figure 1*b*), indicating a significant stress on the lysosomal system in iMGLs.

As phagocytic cells, a properly functioning lysosome is necessary for degradation of engulfed material. Dozens of hydrolytic enzymes that function at low pH are necessary for efficient degradation of cargo. Many of these enzymes are trafficked to the lysosome from the TGN to the lysosome in vesicles chaperoned by mannose-6 phosphate receptors (M6PRs) [41]. The retromer complex has multiple roles in regulating this transport, with studies showing that VPS26a and VPS35 depletion both result in M6PR mis-trafficking and lysosome enlargement [69]. Therefore, one plausible explanation for the enlarged lysosomes observed in SORL1-deficient iMGLs could be mis-trafficking of lysosomal hydrolases because the SORL1–VPS26a–M6PR complex is disrupted. In addition to trafficking and degradation of cargo, microglia depend on lysosomes for a variety of other important cellular activities, the dysfunction of which have been linked to AD including maintenance of homeostasis, lipid metabolism and immune signalling activity [70]. The endo-lysosome itself is a site for immune response signalling. Intracellular Toll-like receptor (TLR) receptors recognize pathogen and damage associated molecular pattern molecules and function in the endo-lysosomal compartment, thus avoiding recognition of self-molecules in the cytosol. Their activities are dependent on functioning hydrolases [71] and both TLR7 and TLR9 require proteolytic cleavage in the endolysosome [72] to form a functioning receptor. Given the critical role of ELN function in the diverse activities of microglia, it is possible that compromised lysosomal function due to downstream effects of SORL1 deficiency leads to alterations in microglial inflammatory states. For example, lysosome instability leads to release of contents into the cytosol activating the NLRP3 inflammasome and release of inflammatory cytokines [73]. Thus, SORL1-retromer dysfunction could have implications for microglial capacity to clear pathogenic protein in brain tisue as well as disruption of normal microglial immune responses.

Taken together, SORL1-retromer dysfunction in the ELN can explain two early and significant events in AD: neurodegeneration and neuroinflammation. We can consider the balance of effects at the endosome and lysosome to be tipped towards the recycling pathway in neurons, leading to mis-localization of important synaptic receptors such as GLUA1, which impairs the inherent electrophysiological properties of the cell. On the other hand, in microglial cells, the balance may be tipped toward the retrograde pathway, leading to mis-trafficking of lysosomal hydrolases, lysosomal stress and an impaired immune response (summarized in figure 1*a*). For effective therapeutic development, it is essential that these cellular mechanisms be elucidated in a cell-type specific manner, as AD is not likely a ‘one drug fits all’ disorder. Cell biological studies in human models that recapitulate the scope of genetic risk for developing AD will be necessary in the development of potential disease-modifying therapies.

## 2. Methods

### (a) Cell lines and genome editing

All cell lines described in this work have been previously published [27,28]. Two isogenic clones per genotype (WT and *SORL1*KO) and 9 independent replicates per clone per genotype (*N* = 18 independent replicates) were used for all of the experiments.

### (b) Differentiation of iPSCs into microglia like cells

iPSCs were differentiated into iMGLs as previously described with some modifications [74]. Briefly, iPSCs were plated in mTESR plus medium supplemented with ROCK Inhibitor (Y-27632; # A3008; Apex Bio) on Matrigel (growth factor reduced basement membrane matrix; # 356231; Corning)-coated 6-well plates (#657160; CELLSTAR). To begin hematopoietic progenitor differentiation, these cells were passaged to get a density of approximately 40 colonies per well of a 6-well plate. On day 0, mTESR plus medium was replaced with STEMdiff Hematopoietic Supplement A medium from the STEMdiff Hematopoietic kit (# 05310; STEMCELL technologies). On day 3, when colonies became flattened, medium was replaced with STEMdiff Hematopoietic Supplement B medium from the STEMdiff Hematopoietic kit (# 05310; STEMCELL technologies). Cells remained in this medium for seven additional days. By day 10, non-adherent hematopoietic progenitor cells (HPCs) shedding from the flattened colonies were harvested by removing medium. Any remaining HPCs/floating cells were collected by gentle PBS washes. For experiments, HPCs were plated at a density of 0.4 M cells per well of a Matrigel coated of a 6-well plate in microglia differentiation medium for 25 days. For storage, HPCs were frozen using Bambanker cell freezing medium (#BBH01; Bulldog-Bio). Microglia differentiation medium comprised of DMEM-F12 (#11039047; Thermo Fisher Scientific), Insulin-transferrin-selenite (#41400045; Thermo Fisher Scientific), B27 (# 17504-044; Thermo Fisher Scientific), N2 (# 17502-048; Thermo Fisher Scientific), glutamax (# 35050061; Thermo Fisher Scientific), non-essential amino acids (# 11140050; Thermo Fisher Scientific), monothioglycerol (# M1753; Sigma), Insulin (# I2643; Sigma) freshly supplemented with TGF-β (#130-108-969, Miltenyl), IL-34 (# 200-34; Peprotech) and M-CSF (#PHC9501; Thermo Fisher Scientific). On day 25, this medium was supplemented with CD200 (#C311; Novoprotein) and CX3CL1 (#300-31; Peprotech) for maturation of microglia. Cells remained in this medium for 7 days. On day 32 microglia differentiation was complete and this timepoint was used for all experiments.

### (c) Immunocytochemistry

For immunostaining, Poly-l-lysine hydrobromide (#P6282; Millipore Sigma)-coated coverslips (12 mm diameter, #1760-012; cglifesciences) placed in a 24-well plate were used. Poly-l-lysine coating of coverslips was performed according to manufacturer's instructions. Briefly, 500 µl of 100ug/ml Poly-l-lysine hydrobromide was added to 24-well plates with coverslips, incubation was done for 30 min at room temperature in the cell culture hood and the plates were then washed with sterile water three times and left in the cell culture hood overnight after aspirating water from the last wash. On the next day, iMGLs were plated at a density of 150 000 cells per well of a 24-well plate on glass coverslips coated with 100 ug ml^−1^ Poly-l-lysine hydrobromide. After 2 days in culture, cells were fixed for 10 min in 4% paraformaldehyde in 1× PBS without calcium and magnesium, prepared from 16% formaldehyde solution (#043368.9 M, Thermo Fisher Scientific). Cells were then washed with PBS with 0.05% tween 20 detergent (PBST), permeabilized with 0.1% triton-X 100 for 15 min and incubated in blocking buffer containing 5% goat serum in PBS for 1 h. After blocking, the following primary antibodies were added and cells were incubated overnight at 4°C. Rabbit CX3CR1 (#ab8020; Abcam) at a dilution of 1 : 500 and Mouse LAMP1 (#sc20011; Santa Cruz) at a dilution of 1 : 200. On the next day, cells were washed thrice with PBST and incubated with the following secondary antibodies for 1 h: goat anti-Rabbit Alexa fluor 488 (# A-11034; Thermo Fisher Scientific) and goat anti-Mouse 594 (# A-11032; Thermo Fisher Scientific). Next, cells were washed thrice with PBST and the coverslips were mounted on glass slides (#12-150-543; Thermo Fisher Scientific) using ProLong Gold Antifade Mountant with DNA Stain DAPI (#P36931; Thermo Fisher Scientific).

#### (i) Measurement of lysosome size

All microscopy and image processing were performed under blinded conditions. Confocal *z* stacks were obtained using a Leica TCS SP8 confocal laser microscope and Leica Application Suite X (3.5.5.19976) software. The LIGHTNING adaptive deconvolution (Leica Microsystems) feature in the LAX software was applied to all images and a 63× apochromat oil immersion objective was used for capturing images. To measure lysosome size, 10 images per clone were analysed (2 clones per genotype; 2 WT and 2 *SORL1*KO clones) using the CellProfiler software [75]. AA mMicroglia-specific marker, CX3CR1 channel (green), was used as the mask and LAMP1 positive puncta were identified using automated segmentation algorithms in CellProfiler. The pixel areas of each LAMP1 positive puncta were measured and have been presented as mean areas of all the puncta per image in figure 1*c*.

## Data Availability

This article does not contain any additional data.
